# Cellulose Surface Degradation by a Lytic Polysaccharide Monooxygenase and Its Effect on Cellulase Hydrolytic Efficiency*

**DOI:** 10.1074/jbc.M114.602227

**Published:** 2014-10-31

**Authors:** Manuel Eibinger, Thomas Ganner, Patricia Bubner, Stephanie Rošker, Daniel Kracher, Dietmar Haltrich, Roland Ludwig, Harald Plank, Bernd Nidetzky

**Affiliations:** From the ‡ Institute of Biotechnology and Biochemical Engineering, Graz University of Technology, Petersgasse 12, A-8010 Graz, Austria,; § Institute of Electron Microscopy and Nanoanalysis and; ** Graz Centre for Electron Microscopy, Steyrergasse 17, A-8010 Graz, Austria,; ¶ Department of Food Science and Technology, BOKU-University of Natural Resources and Life Sciences, Muthgasse 18, A-1190 Vienna, Austria, and; ‖ Austrian Centre of Industrial Biotechnology, Petersgasse 14, A-8010 Graz, Austria

**Keywords:** Atomic Force Microscopy (AFM), Biofuel, Cellulase, Cellulose, Copper Monooxygenase, GH61-AA9, Lytic Polysaccharide Monooxygenase (LPMO), Oxidative Cellulose Surface Degradation, Synergy

## Abstract

Lytic polysaccharide monooxygenase (LPMO) represents a unique principle of oxidative degradation of recalcitrant insoluble polysaccharides. Used in combination with hydrolytic enzymes, LPMO appears to constitute a significant factor of the efficiency of enzymatic biomass depolymerization. LPMO activity on different cellulose substrates has been shown from the slow release of oxidized oligosaccharides into solution, but an immediate and direct demonstration of the enzyme action on the cellulose surface is lacking. Specificity of LPMO for degrading ordered crystalline and unordered amorphous cellulose material of the substrate surface is also unknown. We show by fluorescence dye adsorption analyzed with confocal laser scanning microscopy that a LPMO (from *Neurospora crassa*) introduces carboxyl groups primarily in surface-exposed crystalline areas of the cellulosic substrate. Using time-resolved *in situ* atomic force microscopy we further demonstrate that cellulose nano-fibrils exposed on the surface are degraded into shorter and thinner insoluble fragments. Also using atomic force microscopy, we show that prior action of LPMO enables cellulases to attack otherwise highly resistant crystalline substrate areas and that it promotes an overall faster and more complete surface degradation. Overall, this study reveals key characteristics of LPMO action on the cellulose surface and suggests the effects of substrate morphology on the synergy between LPMO and hydrolytic enzymes in cellulose depolymerization.

## Teaser

**Background:** Lytic polysaccharide monooxygenase (LPMO) has recently been discovered to depolymerize cellulose.

**Results:** Dynamic imaging was applied to reveal the effects of LPMO and cellulase activity on solid cellulose surface.

**Conclusion:** Critical features of surface morphology for LPMO synergy with cellulases are recognized.

**Significance:** Direct insights into cellulose deconstruction by LPMO alone and in synergy with cellulases are obtained.

## Introduction

Plant cell wall cellulose is the most abundant carbohydrate material in nature. It is built from linear polysaccharide chains of several hundreds of β-1,4-linked d-glucosyl units. Multiple chains are organized spatially into a densely packed crystalline material (1, 2). More disordered parts of the cellulose, which are also present in natural or technologically processed substrates, are usually called amorphous. In contrast to amorphous regions, crystalline cellulose is highly resistant to degradation. The low efficiency of its conversion threatens the commercial viability of biofuels or base chemicals produced from cellulosic biomass (3, 4).

**FIGURE 1.**
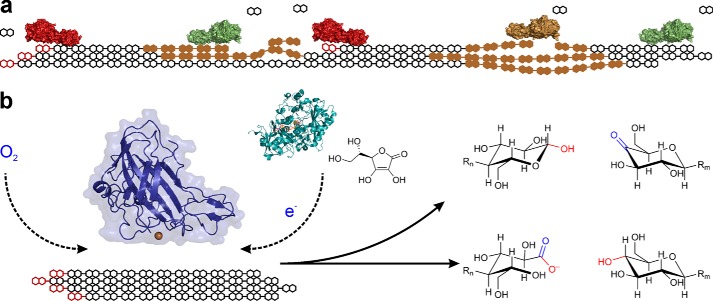
*a*, hydrolysis of crystalline-ordered and amorphous-unordered (*brown*) cellulose is catalyzed by a typical set of fungal cellulases, including chain end-cleaving cellobiohydrolases (CBH I, *red*; CBH II, *green*) and internally chain-cleaving endoglucanase (*brown*). Linear cellulose chains are represented by their cellobiosyl-dimer units, with the reducing end shown in *red*. Cellobiose is the main soluble sugar produced by the cellulases. *b*, oxidative O_2_-dependent attack of LPMO on the cellulose surface, resulting in internal chain cleavages and release of C1′ or C4 oxidized oligosaccharides harboring one oxygen atom from the O_2_ oxidant (indicated in *blue*) (18–20). C1′ oxidation produces a 1,5-lactone product that hydrolyzes rapidly to give a d-gluconic acid moiety, which is shown. LPMO contains surface-exposed catalytic copper (*brown sphere*) that is required for activity (12, 14, 16). The structure of GH61D (PDB code 4B5Q) from *Phanerochaete chrysosporium* is used for depiction (21). Electrons required in the LPMO reaction *in vitro* can be delivered from the flavo-heme protein cellobiose dehydrogenase or from a small-molecule reductant such as l-ascorbic acid (12, 14).

Cellulases represent the current paradigm for enzymatic cellulose degradation through hydrolytic depolymerization (1, 4). Cellulases comprise internally chain-cleaving endoglucanases (EG)^4^ and processively chain end-cleaving cellobiohydrolases (CBHs) as their main activities (1). Different cellulolytic activities operate synergistically; that is, combinations of enzymes are hydrolytically more efficient than expected from the sum of their individual activities (5–7). Cellulase synergism originates from the dynamic interplay between endo- and exo-modes of cellulose chain cleavage as well as from complementary enzyme specificities for degrading crystalline and amorphous cellulose material (Fig. 1*a*) (6–9). Cellulases adsorb strongly to cellulose (7, 10), and their combined action on the solid surface is reflected by strong three-dimensional degradation where amorphous material is degraded at higher rates than crystalline structures (8, 11).

Originally discovered for its activity on chitin (12, 13), lytic polysaccharide monooxygenase (LPMO) employs a unique principle of cellulose degradation utilizing C-H activation followed by O_2_-dependent chain cleavage (12, 14–16). LPMO is a mononuclear type II copper enzyme (14, 17) that requires an external electron donor, a small molecule, or a partner redox-protein for activity (12, 14, 18). Glycosidic bond cleavage in cellulose involves oxidation of C1′ or C4 of an intrachain cellobiosyl moiety (Fig. 1*b*) depending on the type of LPMO used (19, 20). A common feature of LPMO structures is a flat protein face with the active site located near its center and the catalytic metal exposed outward (14, 20–23). Interaction of LPMO with cellulose surfaces is, therefore, thought to occur via the flat protein face (12). Oxidative chain cleavages in crystalline areas of the substrate are expected to cause local disruptions of the ordered cellulose structure (12, 24, 25). Substrate decrystallization effects might, therefore, facilitate hydrolytic cellulose depolymerization. LPMO is, therefore, believed to have significant potential of enhancing the efficiency of cellulosic biomass conversion (17, 25–27), and a number of studies have shown that LPMO does act in synergy with cellulase activities (5, 26, 28).

Current understanding of cellulose degradation by LPMO is limited by the absence of immediate and direct evidence on the enzyme action on the solid surface of the substrate. LPMO activity has been demonstrated from the slow release of oxidized soluble cello-oligosaccharides (19, 20, 27), but the proposed chemical and structural disruption of the cellulose surface has not been demonstrated so far. Thinking of how LPMO might act alone and in synergy with cellulases in insoluble cellulose degradation is, therefore, mostly hypothetical and has so far been inferred only indirectly from protein structures and biochemical data on soluble product formation from cellulosic substrates. The present study provides relevant insight based on so far elusive direct evidence. LPMO caused changes in the surface morphology of a mixed amorphous-crystalline cellulosic substrate at spatial and temporal resolution were measured, and their effects on subsequent surface degradation by cellulases were uncovered. A refined view of the action of LPMO on the cellulose surface shows the possibility for synergy but also for competition with cellulases in crystalline cellulose degradation. Morphological targets for synergy between LPMO and cellulases were thus identified on the cellulose surface.

## EXPERIMENTAL PROCEDURES

### 

#### 

##### Materials

Unless stated, standard chemicals were of the highest purity available from Carl Roth (Karlsruhe, Germany).

##### Enzymes

Purified LPMO from *Neurospora crassa* (LPMO-03328 gene product (NCBI accession number XP_955892)) was prepared by a reported protocol (29). Complete *Trichoderma reesei* cellulase was from fungal culture (strain SVG17) on wheat straw (30, 31). CBH I was purified from the complete *T. reesei* cellulase via a modified ion exchange protocol as described elsewhere (32). Purified *T. reesei* CBH II was recombinant enzyme preparation from *Pichia pastoris* (33). EG (from *Trichoderma longibrachiatum*) was from Megazyme (Dublin, Ireland) and used without further purification. β-Glucosidase from *Aspergillus niger* was from Megazyme.

##### Protein Determination

Molar extinction coefficients for individual enzymes in solution were determined from the protein sequence from UniProt using ProtParam or were taken from literature (ϵ_CBH I (P62694) = 86,760 m^−1^cm^−1^; ϵ_CBH II (P07987) = 97,665 m^−1^cm^−1^; ϵ_EG (Q12714) = 74,940 m^−1^cm^−1^; ϵ_LPMO-03328 = 51,130 m^−1^cm^−1^).

##### Cellulosic Substrates

Avicel PH-101 was from Carl Roth. A mixed amorphous-crystalline cellulosic substrate (MACS) was prepared from Avicel PH-101 using partial dissolution in and regeneration from the ionic liquid 1-butyl-3-methylimidazolium chloride as described elsewhere (30, 34, 35). The absence of ionic liquid in the final substrate preparation was confirmed by mass spectroscopy and simultaneous thermal analysis, as described in an earlier study (34). The MACS substrate presents an amorphous cellulose matrix in which cellulose crystallites of different size are distributed in an irregular fashion (8, 30, 34). Nanocrystalline cellulose was prepared from Whatman® (Sigma) qualitative filter paper according to a protocol from literature (36).

##### Enzymatic Reactions

Experiments were done at 1.0 mg cellulose ml^−1^ in 50 mm sodium phosphate buffer, pH 6.0, using a total volume of 500 μl in static Eppendorf tubes sealed with oxygen-permeable Parafilm. Each experiment was done in independent triplicates using an individual Eppendorf tube for each sampling point. l-Ascorbic acid was present at 7.5 μm. Enzyme loadings (μg of protein mg^−1^ cellulosic substrate) were as follows: LPMO (9), complete cellulase (25), CBH I (100), CBH II (100), and EG (100). β-Glucosidase was added to each reaction at a loading of 5 μg mg^−1^ cellulosic substrate.

Synergy between LPMO and cellulases was examined in two different assays that are referred to as “sequential” or “simultaneous.” In the sequential assay, which involved pretreatment of substrate with LPMO before the action of cellulases, the used cellulosic substrate was incubated at 25 °C with LPMO. The negative control did not contain LPMO or contained LPMO lacking l-ascorbic acid. The total liquid volume was 450 μl. Incubation was for 5 h in all reactions except for 12 h for the reaction where complete cellulase was added in the second step. Reaction tubes were heated to 50 °C, and 50 μl of hydrolytic enzyme solution (complete cellulase, CBH I, CBH II, or EG) were added. Samples were withdrawn every 15 min up to 1 h. For sampling, one of the reaction tubes was removed, and enzyme was inactivated at 95 °C for 10 min. Solid material was removed by centrifugation at 9300 × *g* for 1 min at 4 °C. The cleared supernatant was used for soluble product analyses.

In the simultaneous assay, which did not involve substrate pretreatment by LPMO, cellulosic substrate was incubated at 50 ° C with LPMO and CBH I, both added at the same time. β-Glucosidase was also present. The total reaction volume was 500 μl. The negative control did not contain LPMO or contained LPMO lacking l-ascorbic acid. Samples were withdrawn after 5 and 96 h and were processed as described above for the sequential assay.

##### Fluorescence Dye Adsorption Analyzed with Confocal Laser Scanning Microscopy (CLSM)

MACS substrate was incubated with LPMO for 12 h as described above. Enzyme was removed by washing with absolute ethanol followed by repeated washing with Milli-Q water. Sample was equilibrated for 2 h in staining buffer (2.5 mm MgCl_2_·6H_2_O, 16 mm (NH_4_)_2_SO_4_, 67 mm Tris-HCl, pH 8.4) and then transferred into an aluminum foil-covered reaction tube with 10 ml of freshly prepared staining buffer that contained 5 μm SYTO-62 (Invitrogen). Staining was done for 12 h at 4 °C followed by dye removal through extensive washing with staining buffer. Sample was stored in staining buffer under exclusion of light until CLSM analysis within maximally 4 h. Negative controls were prepared in exactly the same manner except that no SYTO-62 staining was used, no LPMO was applied, or LPMO without l-ascorbic acid was applied.

Sample analysis was performed with a Leica TCS SPE confocal laser microscope (Leica Microsystems, Wetzlar, Germany). Samples were excited with a 635-nm laser beam, and emitted light was detected in the range 645–709 nm. At the same time, transmission images of the transparent samples were obtained at 488 nm. Photomultiplier gain, offset, and Z-step size were carefully optimized for each channel to provide the best signal/noise ratio possible. Confocal stacks were acquired with a Leica ACS APO × 63 OIL CS objective (NA: 1.30). Image analysis and background subtraction was performed using ImageJ 1.47v (rsbweb.nih.gov).

##### Atomic Force Microscopy (AFM) Imaging

A commercial Dimension 3100 Hybrid AFM operated by a Nanoscope 4a Controller (Bruker Nano Surface Offices, Santa Barbara, CA) was used in Tapping Mode (liquid). The instrument was equipped with a liquid probe holder and OMCL-RC800PSA cantilevers (Olympus Probes, Tokyo, Japan). Experiments were performed at ∼20 °C using a laboratory-built liquid cell (8, 30). Scan rates, set points, and drive amplitudes were observed continuously to ensure stable measurement conditions and to prevent image convolution by tip-related artifacts along with lowest possible energy dissipation on the sample. Areas of interest on the specimens were carefully chosen based on light microscopy images, and a series of reference images (*N* ≥ 5) was recorded to ensure the homogeneity of the substrate surface. For time-resolved AFM imaging with quantitative evaluation of surface volume degradation, it was important to select an area of the substrate surface that contained a large cellulose crystallite, which because of its very slow enzymatic degradation served as a height marker to follow degradation of the surrounding material.

Before the AFM experiments, the MACS was treated as described previously (8, 30) to prepare a nano-flat surface and mounted in the liquid cell, and reference images were taken before the injection of the enzyme solution. The cellulose substrate concentration was 0.50 mg ml^−1^, and enzyme loadings were as described above. Enzymatic reactions were followed at 20 °C in a continuous manner for typically 5 h.

AFM image processing and analysis was performed using Gwyddion 2.31 (released 02/21/2013) and Nanoscope (Build R3Sr4.94136, Bruker Nano Surface Offices). Data analysis was performed using Origin 8.5 (OriginLab Corp., Northampton, MA).

##### Analytics

d-Glucose was determined colorimetrically with glucose oxidase and peroxidase (37). Cellobiose and higher oligosaccharides were analyzed with high performance anion exchange chromatography coupled to pulsed-amperometric detection (HPAEC-PAD) (Dionex BioLC, Thermo Fisher Scientific, Waltham, MA) as described elsewhere (30). Soluble products of LPMO were also analyzed by HPAEC-PAD. Briefly, a CarboPac® PA200 column (3 × 250 mm) and a CarboPac® PA200 guard column (3 × 50 mm), both from Thermo Fisher Scientific, were used. Temperature was 30 °C, and the flow rate was 0.4 ml min^−1^. The column was equilibrated with 120 mm sodium hydroxide for 5 min. Samples were analyzed with a linearly ascending gradient in sodium acetate (up to 250 mm). The identity of d-gluconic acid was assigned using authentic standard. Identification of other products was not pursued.

## RESULTS AND DISCUSSION

**FIGURE 2.**
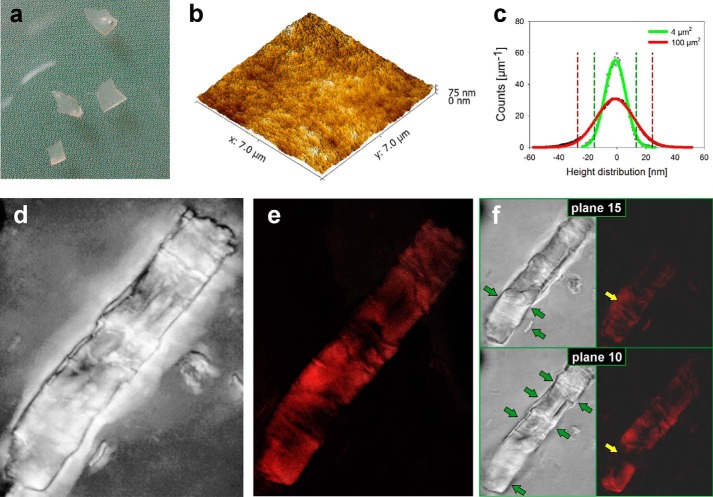
**LPMO is specific for oxidizing crystalline cellulose surfaces and does so by introducing carboxylic acid groups.** *a*, visual appearance of the mixed amorphous-crystalline cellulose substrate prepared from Avicel PH-101 using partial dissolution in and regeneration from ionic liquid. *b*, AFM height image of the substrate surface in a ultramicrotome-cut cellulose sample. *c*, height distribution analysis reveals the nano-flat character of the substrate surface. *d*, maximum intensity projection of the recorded transmission microscopic images of a substrate area where a large cellulose crystallite is embedded in an amorphous matrix. The crystallite is a remnant from the original Avicel PH-101 not dissolved during ionic liquid treatment. It is composed of cellulose allomorph I, as shown by XRD (33). *e*, maximum intensity projection of the recorded fluorescence signal from the same substrate area after incubation with LPMO (9 mg g^−1^; supplemented with 7.5 μm l-ascorbic acid; 25 °C; 12 h) and subsequent staining for 12 h with 5 μm SYTO-62, a small molecule fluorescent probe for carboxyl groups (38). The figure shows that crystalline surfaces are preferentially labeled with the fluorescent dye after substrate incubation in the presence of LPMO. This suggests preferred attack of LPMO on these surfaces. *f*, transmission microscope images (*left panel*) with the corresponding fluorescence images (*right panel*) from two focal planes separated by a vertical distance of 4 μm. Focused specimen parts, which are recognized clearly by detailed structures in the transmission images (indicated with *green arrows*), correspond to the surface of the cellulose crystallite. CLSM images reveal that the SYTO-62 fluorescence signal is confined exclusively to crystalline parts in focus over different planes (*yellow arrow* in image in *plane 15*) and that the fluorescence signal is not present at interior parts of the crystallite (*yellow arrow* in image in *plane 10*). Therefore, these images show that LPMO action is restricted to the surface of the cellulose crystallite. Suitable controls showed that untreated cellulose crystals or cellulose crystals treated with LPMO in the absence of l-ascorbic acid did not become fluorescent when stained with SYTO-62. Fluorescence images in (*e* and *f*) are background-subtracted and contrast-enriched. *Scale bars*, 10 μm.

The LPMO used in this study was the LPMO-03328 gene product from the fungus *N. crassa*. In the sequence-based classification of LPMOs among the carbohydrate-active enzymes (14), the enzyme belongs to Auxiliary Activity family 9 (AA9, formerly GH-61). Also based on sequence similarity (18, 19) and shown biochemically (19), the LPMO used was further categorized into the group of C1-oxidizing LPMOs. Highly purified recombinant enzyme was obtained from *P. pastoris* expression culture as described previously (29). A specially designed model substrate, providing amorphous and crystalline regions alternating on a nano-flat cellulose surface (Fig. 2, *a–c*) (8, 30, 34) was key in the characterization of the enzymatic degradation process catalyzed by the LPMO. Effects of LPMO action on cellulose surface chemistry and structure were, therefore, measured for the first time. A fundamentally new LPMO assay based on adsorption of the fluorescent dye SYTO-62, originally developed for measurement of carboxyl groups on polymer bead surfaces (38), was adapted here to identify and localize LPMO-catalyzed formation of carboxyl groups on the cellulose surface. Analysis by CLSM provided spatial resolution of the fluorescence intensities, thus allowing for characterization of the specificity of the LPMO for attacking cellulose surface material. Lateral and vertical degradation activities of the LPMO could also be measured. Time-resolved *in situ* AFM in liquid environments was used to measure changes in surface topology and material character caused by LPMO action.

### 

#### 

##### LPMO-catalyzed Cellulose Surface Oxidation Revealed by Using Fluorescence Dye Adsorption

Cellulose model substrate was incubated with LPMO, and the fluorescence dye adsorption assay was applied for subsequent surface characterization. Fig. 2, *d–f*, shows transmission/fluorescence microscopic images of LPMO-treated and then SYTO-62-stained substrate. A strong increase in SYTO-62 fluorescence from the cellulose surface after incubation with enzyme under turnover conditions in the presence of l-ascorbic acid provided a clear indication for carboxyl group formation by the LPMO (Fig. 2*e*). Suitable controls (see “Experimental Procedures”) did not show increased surface fluorescence after treatment with SYTO-62 (data not shown). Interestingly, fluorescence signals were detected almost exclusively from crystalline surface areas of the cellulose, as shown in Fig. 2, *e–f*, demonstrating high selectivity of the LPMO for oxidizing just the ordered substrate structures. Using CLSM, we showed that SYTO-62 fluorescence was emitted from the outermost surface of the crystalline material, whereas subjacent layers of the crystalline cellulose were clearly non-fluorescent (Fig. 2*f*). Therefore, the LPMO activity appeared to have caused only minimal oxidative substrate degradation in the vertical direction. Fig. 2*e* also reveals that the crystalline cellulose surface was oxidized in large patches of up to 1500 μm^2^, consistent with surface degradation through multiple site attacks by the LPMO.

##### Cellulose Surface Degradation by LPMO Monitored with AFM

**FIGURE 3.**
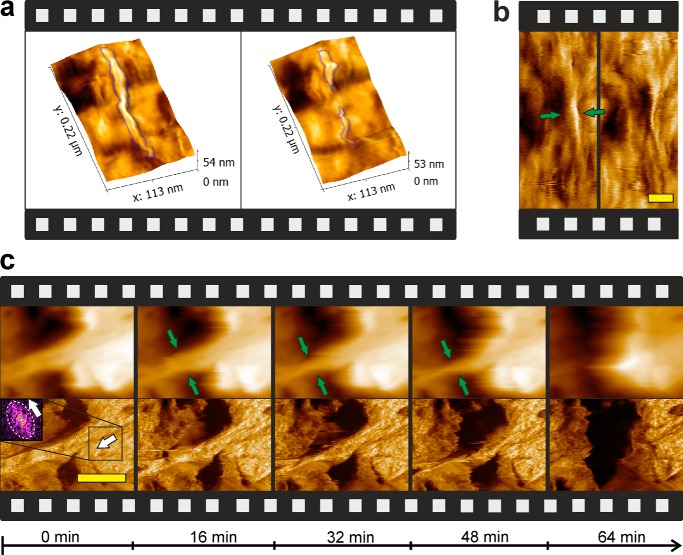
**AFM imaging of LPMO action on the cellulose surface and its effect on CBH I activity.** *a*, three-dimensional surface representation overlaid with phase information to show the attack of LPMO (9 mg g^−1^ substrate; 20 °C; 12 h) on a cellulose fibril (*blue outline*) in the *middle* and from the *top*, thus causing a large amount of internal degradation. *b*, phase images showing fibril degradation by LPMO through major thinning from the sides, as indicated by *green arrows*. AFM images show substrate before and after 12 h of incubation with LPMO supplemented with 7.5 μm l-ascorbic acid as reductant. Amorphous material is visualized by a dark color in phase or phase-overlaid images. It was resistant against degradation by LPMO. *Scale bar*, 30 nm. *c*, time-resolved AFM sequences (height images on *top*, phase images on *bottom*) showing surface degradation by CBH I on a substrate preincubated with LPMO. Gradual degradation of a crystalline surface feature is observed as a result of thinning from the top and from the sides, as indicated by *green arrows*, eventually leading to complete dissolution of a large set of fibril bundles. Fiber orientation on the crystalline surface is demonstrated by 2D Fast Fourier Transformation analysis (embedded in Gwyddion 2.31) of the *highlighted rectangle*. Deviation from a circular symmetric to an ellipsoid spectrum (*white dotted envelope*) shows the fiber orientation by a 90° rotation (*white bold arrows*). *Scale bar*, 300 nm.

Time-resolved AFM data were obtained from LPMO-catalyzed surface degradation of the nanoflat cellulosic substrate. Results showed that LPMO promoted cellulose surface structure degradation to only a small degree. The overall volume degradation of surface material was minimal. However, enzyme activity was detected clearly on crystalline regions of the substrate, which were removed at a slow rate (Fig. 3, *a* and *b*). Regions of surrounding amorphous material were not affected. Interestingly, small crystalline nano-fibrils, present in large number on the cellulose surface where they were irregularly distributed within otherwise predominantly amorphous material, were degraded effectively by the LPMO, as also shown in Fig. 3, *a* and *b*. Fibril degradation proceeded primarily by thinning, starting from the side walls, but also by cleavage in the middle to create smaller and shorter fragments. Discrete crystalline surface structures were thus dissolved as a result of the action of the LPMO. Even though formation of oxidized oligosaccharides was detectable in solution (*e.g.* d-gluconic acid), the released amount was too small for exact quantification. It seems, therefore, that activity of the LPMO used was captured not only more directly but also more sensitively through analysis of the oxidative surface degradation that it produced.

##### Synergy between LPMO and Hydrolytically Cellulose-degrading Enzymes

We proceeded by examining the synergy effects between LPMO and hydrolytic enzymes. Cellulose pretreated with LPMO (sequential assay) was used as the substrate for cellulases, and the resulting surface degradation was analyzed with *in situ* AFM. Comparison of time-resolved AFM sequences for conversion of LPMO treated and untreated substrate revealed a basis for cooperativity between oxidative and hydrolytic enzymes depending on the morphology of the cellulose surface. Because no cellulose specimen is the exact replica of another, comparative analysis is possible to the extent that highly characteristic features of the surface degradation are worked out. AFM observations reported recur within different areas of a single sample and are fully reproducible across different substrate specimen.

In a recent AFM study we characterized the specificities of the major fungal cellulases (EG, CBH I, CBH II) for surface material degradation in the same cellulose substrate that was now used to investigate the action of LPMO (8, 30). EG and CBH II degrade amorphous material (8, 30), and we found here that neither enzyme takes advantage of substrate pretreatment with LPMO in terms of enhanced rate or completeness of the overall surface degradation. Crystalline material not attacked in untreated substrate (small fibrils, large crystallites) remained completely resistant to EG or CBH II activity after the incubation with LPMO. The initial rate of soluble sugar release by each of the two cellulases was also unaffected within error limit by substrate treatment with the LPMO. Considering studies that state up to 2-fold LPMO-caused stimulation in cellulose conversion by different EG enzymes or by CBH II (17, 28), it is important to emphasize key differences in the ways LPMO-cellulase synergy was assayed herein and in several other studies.

Our sequential assay involves defined preincubation of cellulosic substrate with the LPMO under established conditions for lytic oxidation (29) and defines synergy with the cellulase in question as the ratio of initial rates of reducing sugar release from LPMO-treated and untreated substrate in the second step of the overall oxidative-hydrolytic cellulose depolymerization. The simultaneous assay used in several earlier studies (5, 26, 28, 39) and also herein (see later) measures the enhancement of soluble sugar release (typically after a long incubation time of several days) caused by supplementation of the cellulase with LPMO. The reason to prefer the sequential assay here as our main analytical tool was that it rigorously evaluates the immediate effect of substrate transformation by the LPMO (see Fig. 2, *e* and *f*, and Fig. 3, *a* and *b*) on the cellulase activity, whereas it excludes potential ambiguity of the simultaneous assay where LPMO and cellulase can influence each other in various ways in their activity and also stability. Finally, sequential actions of LPMO and cellulases might be just as relevant biologically as is their simultaneous action.

Being mainly active on crystalline cellulose (1, 40), CBH I complements EG and CBH II in their substrate specificity. However, in the mixed amorphous-crystalline cellulosic substrate used herein, surface degradation by CBH I is measurable only on exposed small fibrils, whereas larger crystalline parts are not degraded within limits of detection, just as the amorphous material (8, 30). Interestingly, therefore, prior action of the LPMO made some of the larger crystalline areas of the substrate clearly accessible for the degradation by CBH I. The time-resolved AFM sequences in Fig. 3*c* reveal gradual degradation of a prominent large-sized and elongated crystalline feature of the substrate, where the enzymatic attack took place primarily through thinning from the sides, and complete dissolution occurred within ∼1 h of incubation. Nearby crystalline material also showed clear indications of progressing degradation with incubation time. In untreated substrate incubated with CBH I, degradation of similarly sized crystalline cellulose structures has never been observed, as already reported in previous papers (8, 30) and also confirmed in this study by investigating multiple substrate samples (*N* ≥ 5).

**FIGURE 4.**
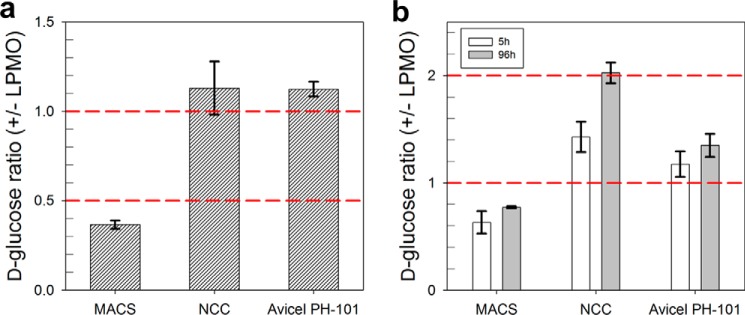
**Synergy between LPMO and CBH I during degradation of different cellulosic substrates determined in sequential (*a*) and simultaneous (*b*) assays.** The substrates used were MACS, nanocrystalline cellulose (*NCC*), and Avicel PH-101. For the sequential assay (*panel a*), LPMO (9 μg ml^−1^) was incubated with either cellulose preparation (1 mg ml^−1^) in 50 mm sodium phosphate buffer, pH 6.0, for 5 h at 25 °C. l-Ascorbic acid (7.5 μm) was present as the reductant. The total volume was 500 μl, and incubations were done in static Eppendorf tubes. CBH I (100 μg ml^−1^) supplemented with β-glucosidase (5 μg ml^−1^) was added to the reaction mixture, and incubation was continued at 50 °C for 1 h. The simultaneous assay (*panel b*) used identical conditions except that all reactants were already present at reaction start. Temperature was 50 °C, and reaction time was 96 h. The d-glucose concentration was measured with an enzymatic assay. It was confirmed using analysis with high performance anion exchange chromatography with pulsed amperometric detection that d-glucose accounted for ≥99% of the total soluble products present in the supernatant under these conditions. The measured data are plotted as ratio of sugar formed in reactions containing or lacking LPMO. A value exceeding unity, therefore, indicates a synergistic effect. A value below unity indicates that preincubation with LPMO (*panel a*) or the presence of LPMO (*panel b*) results in apparent inhibition of the d-glucose release by CBH I. Results are shown in *bars* with S.D. from the three independent experiments indicated.

The herein discovered synergy between LPMO and CBH I in the degradation of highly recalcitrant cellulosic material (*i.e.* large crystallites) is, however, not reflected by a boost in the rate of reducing sugar formation from substrate pretreated with LPMO. On the contrary, as shown in Fig. 4*a*, hydrolytic activity of CBH I was lowered significantly (∼3-fold) on LPMO-treated as compared with untreated cellulose, suggesting inhibition effects rather than synergy between LPMO and CBH I during degradation of the particular cellulosic substrate used. The apparent conflict in these findings (Figs. 3*c* and 4*a*) is resolved considering that LPMO-catalyzed oxidative degradation of nano-fibrils on the cellulose surface (Fig. 3, *a* and *b*) could have removed or rendered inaccessible the major substrate material for hydrolysis by CBH I. Larger crystalline features, identified from AFM data as major sites of synergy between CBH I and LPMO (Fig. 3*c*), accounted for only a small part (≤5%) of the total available surface area in the substrate used so that in the overall hydrolysis measured as the release of soluble sugars, competition between the two enzymes appears to have outweighed their locally observed synergistic interaction.

We also found, again based on measurement of soluble sugar formation, that LPMO did not exhibit synergy with CBH I in the degradation of isolated crystalline nano-fibrils (Fig. 4*a*). On Avicel PH-101, which despite its poorly defined morphology is a widely used model substrate for highly crystalline cellulosic material (10, 41, 42), a small but significant stimulation (1.1-fold) of the soluble sugar release rate by CBH I was observed when the substrate had been preincubated with LPMO (Fig. 4*a*). Therefore, these results suggest that crystalline cellulosic material presents mesoscopic (that is, above the nano-fibril size) morphological targets for cooperativity between LPMO and CBH I. Local disruption of larger crystalline cellulose surfaces due to oxidation events (Fig. 2, *e* and *f*) might be important to allow for a facilitated attack of CBH I (Fig. 3*c*).

Considering previous work done on the synergy between oxidative-lytic and hydrolytic cellulose-degrading enzymes (5, 26), we also performed simultaneous assays of the combined cellulolytic action of LPMO and CBH I in order to be able to put our results into useful context with literature. Moreover, use of the two enzymes together was also meant to ensure that potential substrate limitation to CBH I caused by prior action of the LPMO was excluded. Using the three cellulosic substrates applied before, release of d-glucose was determined after 5 and 96 h under conditions where CBH I was used in combination with the LPMO or alone. The degree of synergy is expressed as the ratio of the two d-glucose measurements at each time, and the results are summarized in Fig. 4*b*.

Clear lack of synergy between LPMO and CBH I acting on the mixed amorphous-crystalline cellulosic substrate was confirmed from results of the simultaneous assay: CBH I acting alone was ∼1.6-fold more efficient in producing d-glucose at 5 h than the two-enzyme mixture containing LPMO. To the best of our knowledge, turnover frequency of LPMO on crystalline cellulose has not been determined. However, there is good evidence of a relatively slow action of the enzyme (26, 28, 43), one that appears to be minimally an order of magnitude slower than the known action of CBH I on crystalline cellulose (44–46). Therefore, the result in Fig. 4*b* might imply that LPMO and CBH I compete for the same binding (adsorption) sites in the cellulosic substrate used or that only a small amount of cellulose surface degradation by the LPMO is sufficient to achieve significant inhibition of the CBH I. There was a large excess of CBH I (1.54 μm) over LPMO (0.38 μm) present in the reaction, making it somewhat unlikely that LPMO could have outcompeted CBH I from binding to its reaction sites on the cellulose surface. Interestingly, the negative effect of LPMO on d-glucose release was mitigated significantly at longer incubation times, perhaps indicating that substrate material initially less accessible to CBH I when LPMO was present was degraded by CBH I in a later phase of the hydrolysis.

Using nanocrystalline cellulose and Avicel PH-101 as substrates, we observed that, somewhat contrary to results obtained with the sequential assay where synergy between LPMO and CBH I was detectable only to a very small degree (Fig. 4*a*), simultaneous action of the two enzymes gave a substantial stimulation of d-glucose release compared with CBH I acting alone. The synergistic effect was particularly strong when using nanocrystalline cellulose, as seen in Fig. 4*b*. Generally, synergy was increased at long incubation time. With Avicel PH-101 at 5 h, the degree of synergy measured in simultaneous and sequential assays was, however, similar. We are careful in trying to relate cause to effect in respect to enzyme synergy on the nanocrystalline material. Although it is tempting to speculate that LPMO-catalyzed oxidative surface degradation has facilitated the hydrolysis of this highly recalcitrant cellulosic substrate by CBH I, through generation of new free chain ends, for example, one must also consider the possibility of secondary (indirect) effects of the LPMO action. One such effect potentially relevant for hydrolytic degradation of the nanocrystalline cellulose used herein is that the relatively high tendency of the cellulose nanofibrils to form aggregates from colloidal suspension is reduced significantly upon the introduction of additional surface carboxylate groups by LPMO activity. Charged surface groups are known from literature to strongly stabilize colloidal suspensions of nanocrystalline cellulosic material (36, 47, 48).

In summary, synergy between LPMO and CBH I on predominantly crystalline cellulose substrates was demonstrated, in agreement with previous findings in literature (17, 28). Apparent inhibition of CBH I activity on mixed amorphous-crystalline cellulose by prior action of LPMO (sequential assay; Fig. 4*a*) or by the presence of LPMO (simultaneous assay; Fig. 4*b*) is ascribed to the particular nature and composition of the surface of this cellulosic substrate (Fig. 3), and plausible interpretation of the findings was obtained using real-time AFM imaging combined with kinetic analysis of soluble sugar release.

##### Synergy between LPMO and the Complete T. reesei Cellulase System

**FIGURE 5.**
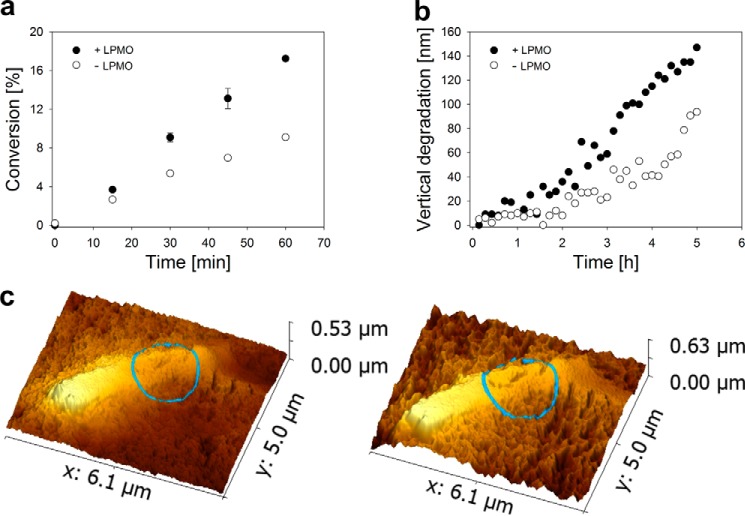
**Effect of substrate pretreatment with LPMO on activity of the complete *T. reesei* cellulase for degrading mixed amorphous-crystalline cellulose analyzed in supernatant (*a*) and directly on the cellulose surface (*b* and *c*).** *a*, LPMO (9 mg g^−1^) was incubated with either cellulose preparation (1 mg ml^−1^) in 50 mm sodium phosphate buffer, pH 6.0, for 12 h at 25 °C. l-Ascorbic acid (7.5 μm) was present as the reductant. Cellulase (25 mg g^−1^) supplemented with β-glucosidase (5 mg g^−1^) was added to the reaction mixture, and incubation was continued at 50 °C for 1 h. The d-glucose concentration was measured enzymatically. It was confirmed using analysis with HPAEC-PAD that d-glucose accounted for ≥99% of the total soluble products present in the supernatant under these conditions. Percent conversion is calculated from the anhydroglucose release. Symbols show measured data, and *error bars* show S.D. from three independent experiments. Pretreatment of substrate by LPMO boost the cellulase activity by a factor of ∼2. *b* and *c*, experiments in the AFM liquid cell were performed in exactly the same way as described above, except that the temperature of the hydrolysis reaction was 20 °C. *Panel c* shows AFM height images that depict a representative surface area of the substrate after LPMO treatment immediately before the addition of cellulase (*left image*) and after 5 h of hydrolysis reaction (*right image*). A large cellulose crystallite (*bright color*) is seen embedded in amorphous material (*darker color*). A *blue circle* is used to indicate part of the cellulose surface where structural disruptions occur within highly crystalline material. Comparison of the *left* and *right* image reveals that cellulase activity results in strong volume degradation in regions of amorphous cellulose, causing the overall surface to become completely rugged with time. Additionally, there is clear activity in the highlighted crystalline region of the substrate where fissures become larger and generally more distinct with time. It is important to emphasize that activity in the highly crystalline areas has only been observed when substrate was pretreated with LPMO. *Panel b* shows a quantitative analysis of time-resolved AFM sequences that were used to measure vertical surface degradation by the cellulases. Slowly degraded crystallites such as the one seen in *panel c* were used as reference points. Pretreatment of substrate with LPMO enhanced surface degradation by the cellulases by a factor of ∼2. Despite different reaction temperatures used in hydrolysis reactions shown in *panel a* and *panel b*, it is worth noting that synergy factors determined from the measurement of soluble product release and measurement of surface degradation were identical within error limit.

Contrary to the individual hydrolytic enzymes examined, a complete EG- and CBH-containing cellulase system clearly benefited from pretreatment of the substrate with LPMO through substantial (∼2-fold) acceleration of surface volume degradation and soluble sugar release, as shown in Fig. 5 *a* and *b*. AFM data reveal furthermore that prior action of the LPMO enabled the cellulases to degrade crystalline surface structures otherwise extremely resistant to enzymatic conversion. Large crystallites like the one shown in Fig. 3*c* remained completely unchanged during prolonged exposure (≥4 h) to cellulase activity alone. After preincubation with LPMO, however, attack of the cellulases promoted strong crystallite surface erosion. This was reflected by material removal and consequent cavity formation predominantly at the sides but also on the top surface of the cellulose crystallite where small cracks grew larger and became clearly more distinct with time (Fig. 5*c*). LPMO synergy with a dynamically interacting system of EG and CBH enzymes could have multiple causes. Interplay between LPMO and CBH I activity was most probably responsible for a unique surface disruption in large crystallite structures (Fig. 3*c*), as already discussed above. Enhancement of cellulose surface degradation in the vertical direction (Fig. 5*b*) was, however, due to the faster loss of amorphous material (and the small fibrils contained in it) from the substrate after pretreatment with LPMO as compared with the untreated substrate. How LPMO becomes involved in the cooperativity among cellulases to degrade mainly amorphous cellulose is an interesting problem. A suggestion supported by the combined evidence from this study and a previous paper of the authors (8) is that LPMO contributes to the degradation of small crystalline fibrils, which are constantly being uncovered on the cellulose surface due to removal of amorphous material (by EG and CBH II) and which in the absence of LPMO are left to degradation by CBH I alone.

In summary, key characteristics of cellulose surface degradation by an Auxiliary Activity family 9-type C1′-oxidizing LPMO from *N. crassa* are revealed, and critical features of surface morphology for LPMO synergy with individual cellulases are recognized. Dynamic interplay between LPMO and CBH I activity appears to be beneficial for degradation of large, hence highly resistant crystalline substrate structures. The effect of LPMO on cellulose saccharification efficiency by a complete mixture of *T. reesei* cellulases involves substrate morphology as a key parameter. A proposed role of “substrate factors” in determining LPMO-cellulase synergy (4, 5, 12) is strongly substantiated through direct observations from the cellulose surface being degraded.

## Footnotes

4 The abbreviations used are:

EG: endoglucanase
LPMO: lytic polysaccharide monooxygenase
CBH: cellobiohydrolase
AFM: atomic force microscopy
CLSM: confocal laser scanning microscopy
MACS: mixed amorphous-crystalline cellulosic substrate
HPAEC-PAD: high performance anion exchange chromatography coupled to pulsed-amperometric detection.
